# Symptomatic bradycardia associated with topical brimonidine gel (Mirvaso) administration: a case report

**DOI:** 10.1093/ehjcr/ytae329

**Published:** 2024-07-10

**Authors:** Daniel Indrarajan Jesudason, Nageswary Appalanaidu, Asif Khan

**Affiliations:** Department of Cardiology, Queen’s Medical Centre, Nottingham University Hospitals NHS Trust, Derby Road, Lenton, Nottingham, Nottinghamshire NG7 2UH, UK; Department of Cardiology, King's Mill Hospital, Sherwood Forest Hospitals NHS Foundation Trust, Mansfield Rd, Sutton-in-Ashfield, Nottinghamshire NG17 4JL, UK; Department of Cardiology, King's Mill Hospital, Sherwood Forest Hospitals NHS Foundation Trust, Mansfield Rd, Sutton-in-Ashfield, Nottinghamshire NG17 4JL, UK

**Keywords:** Symptomatic bradycardia, Brimonidine, Mirvaso, Drug-induced bradycardia, Pharmacology, Case report

## Abstract

**Background:**

Bradycardia can have a number of different aetiologies, including as a side effect of medications. Brimonidine is a rare, but recognized, cause of bradycardia. Brimonidine is indicated in the treatment of facial erythema in rosacea when given as topical brimonidine gel (Mirvaso). It may also be administered as eye drops for raised intraocular pressure in open-angle glaucoma and ocular hypertension. Brimonidine is an alpha-2 agonist, which if systemically absorbed can present with bradycardia, hypotension, and dizziness. The authors are unaware of any other case reports regarding topical administration of Mirvaso in an adult and symptomatic bradycardia.

**Case summary:**

We present the case of a 78-year-old man with a background of rosacea, benign prostatic hyperplasia, and hypertension who had two separate admissions with symptomatic bradycardia. Electrocardiograms showed sinus bradycardia with AV block first degree, with rate recorded as low as 31 b.p.m. during a syncopal episode. These episodes of symptomatic bradycardia were intermittent and had a temporal association with topical administration of Mirvaso. He had no further episodes of bradycardia on discontinuation of Mirvaso and has remained symptom free for over 6 months.

**Discussion:**

The topical administration of Mirvaso should be avoided to broken or inflamed skin. This is owing to the increased risk of systemic absorption, which as in this case report, may present with bradycardia. This case reiterates the importance of completing a full medication history including all topical and parenteral medications in patients with arrhythmia.

**Learning points**

- Brimonidine is a rare cause of bradycardia.
- Brimonidine gel (Mirvaso) should not be applied to damaged or inflamed skin owing to the increased risk of systemic absorption and side effects including bradycardia, hypotension, and dizziness.

## Introduction

Brimonidine is an alpha-2 adrenergic agonist indicated in the treatment of facial erythema in rosacea when applied as a topical gel, known as Mirvaso.^1^ Alpha-2 adrenergic agonists inhibit noradrenaline release from the presynaptic neurone, which may cause hypotension, bradycardia, and sedation.^2^ The use of Mirvaso causes cutaneous vasoconstriction, which in turn can reduce facial erythema. Brimonidine has other indications for raised intraocular pressure in open-angle glaucoma and in ocular hypertension when administered as eye drops. Brimonidine eye drops lower the intraocular pressure by reducing the production of aqueous humour and increasing uveoscleral outflow. We present a case of symptomatic bradycardia in a 78-year-old gentleman associated with topical administration of Mirvaso for rosacea.

## Summary figure

**Table:** 

Date	Event
Day −120 (before admission)	Patient commenced on topical brimonidine gel (Mirvaso) for suspected rosacea
Day −21	Patient drowsy in mornings 1.5–2 h after application of Mirvaso
Day 0 (date of first admission)	Admitted with symptomatic bradycardia with dizziness
Day 2	Discharged after 2 days of cardiac monitoring with no further episodes of symptomatic bradycardia as an inpatient
Day 4	Readmitted following symptomatic bradycardic episode with syncope
Days 5–7	Three further episodes of symptomatic bradycardia (syncope and prolonged unresponsiveness)
Day 8	Mirvaso stopped
Day 11	Discharged with no further episodes of symptomatic bradycardia
Day 56	7-day outpatient SpiderFlash® monitor confirms no further episodes of bradycardia
Day 180	Seen in clinic and remained asymptomatic for 6 months

## Case report

A 78-year-old gentleman with a past medical history of hypertension, benign prostatic hyperplasia, and suspected rosacea attended hospital having had a 2-day history of intermittent dizziness. He was assessed by paramedics prior to attending the emergency department, who noted that he was in sinus bradycardia, heart rate ranging between 30 and 40 b.p.m., with AV block first degree. He had a similar episode the previous morning, during which his systolic blood pressure was recorded at 70 mmHg.

**Figure 1:**
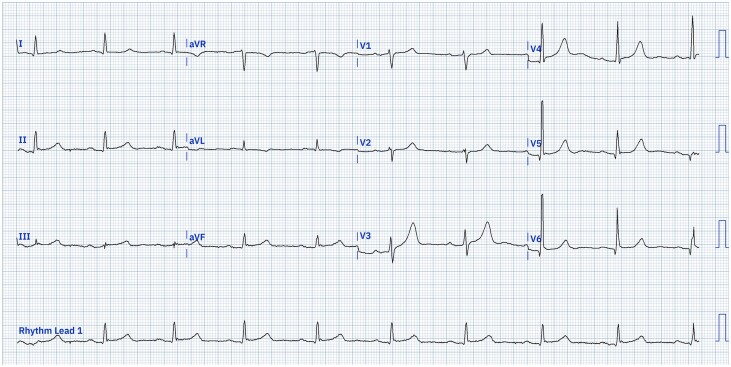
Twelve-lead electrocardiogram from first day of first admission (Day 0). 25 mm/s, 10 mm/mV, sinus bradycardia, heart rate 55 b.p.m., AV block first degree (PR interval 291 ms). ECG digitalized with use of PMCardioApp.

When at hospital, it was noted that his symptoms of bradycardia had resolved without any prior treatment, although his heart rate remained 40–50 b.p.m. (*Figure 1*). Cardiovascular examination was otherwise normal. He was on lisinopril for hypertension, Mirvaso once daily for rosacea and had recently finished a course of oral flucloxacillin for a suspected skin infection of the face. He had been commenced on Mirvaso 4 months prior to admission. He was not on any other medications and had not been on any rate control medications such as beta-blockers previously. Blood tests including full blood count, urea and electrolytes, thyroid function tests, and C-reactive protein were normal. He was reviewed by dermatology who noted erythema, facial swelling, and crustiness over both cheeks as well as dry mouth, but did not feel there was any evidence of infection and Mirvaso was continued. Cardiology review noted he remained in normal sinus rhythm and he was discharged within 2 days. His heart rate had improved to 50–60 b.p.m. with no further episodes of symptomatic bradycardia.

**Figure 2:**
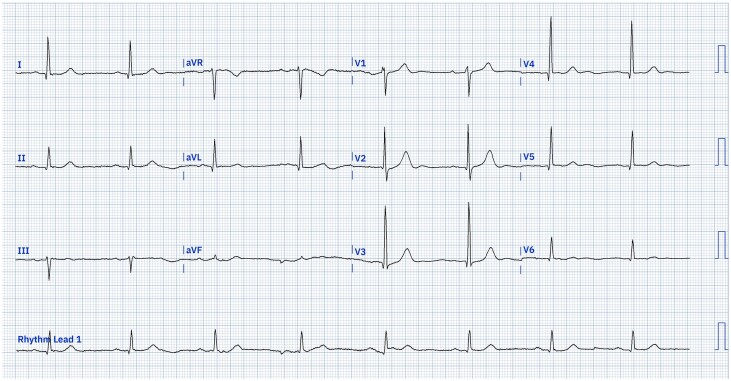
Twelve-lead electrocardiogram from first day of second admission (Day 4). 25 mm/s, 10 mm/mV, sinus bradycardia, heart rate 48 b.p.m., AV block first degree (PR interval 205 ms). ECG digitalized with use of PMCardioApp.

**Figure 3:**
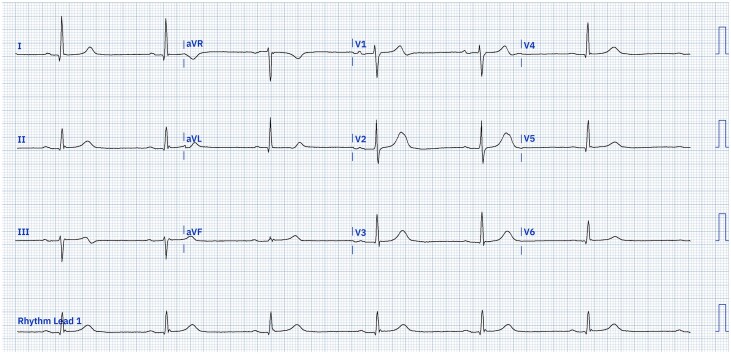
Twelve-lead electrocardiogram from second day of second admission (Day 5) following unresponsive episode. 25 mm/s, 10 mm/mV, sinus bradycardia, heart rate 38 b.p.m., AV block first degree (PR interval 227 ms). ECG digitalized with use of PMCardioApp.

Two days later, he had a syncopal episode at home. It was reported that his unresponsiveness persisted for around 5 min. Emergency services were contacted and his heart rate was recorded at 31 b.p.m. by the paramedic crew. He was given 600 μg of atropine and his heart rate increased to 40 b.p.m. before falling again, requiring a second bolus of 600 μg of atropine. He was also noted to have significant postural hypotension. His heart rate had improved to 40–50 b.p.m. once he attended hospital (*Figure 2*). He was readmitted and was kept on continuous cardiac monitoring whilst an inpatient. During his admission, he was noted to have two further syncopal episodes in the mornings and an episode of prolonged unresponsiveness with hypotension, all whilst sitting (*Figure 3*). The cardiac monitor revealed sinus bradycardia with AV block first degree, during these episodes. There was no evidence of postural hypotension on subsequent checks.

There was concern his presentation may have represented focal seizures. Magnetic resonance imaging of the head was performed, which showed no significant abnormality and neurology review did not feel his presentation was in keeping with seizure activity.

The patient had stated he thought the Mirvaso made his erythema worse. Dermatology review was sought and it was agreed Mirvaso would be stopped (*Summary figure*), and he was continued on alternative topical emollients and steroids.

**Figure 4:**
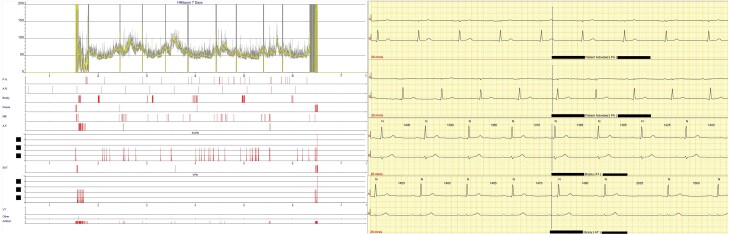
Electrocardiogram traces from 7-day SpiderFlash® monitor including heart rate over 7-day period and patient-activated and auto-triggered sinus bradycardia with heart rate 40–50 b.p.m. (performed on Day 56).

Permanent pacemaker implantation was considered owing to presumed recurrent cardiogenic syncope; however, following discontinuation of Mirvaso, there were no further episodes of symptomatic bradycardia. Further history taking revealed that he typically applied Mirvaso in the mornings, which had a temporal association with the syncopal episodes that he had experienced. He was discharged, had a 7-day outpatient SpiderFlash® monitor, which showed no evidence of significant bradycardia (*Figure 4*), and has had no further episodes of symptomatic bradycardia for over 6 months following Mirvaso being stopped.

## Discussion

The use of Mirvaso is a recognized, but rare cause of bradycardia. The authors are unaware of any other case reports regarding topical administration of Mirvaso in an adult and symptomatic bradycardia. Mirvaso may be used in the treatment of rosacea as it causes cutaneous vasoconstriction, which in turn reduces facial erythema.

The literature outlining the rare side effect of bradycardia from preparations of brimonidine including Mirvaso in adults is relatively sparse. A case report describes the intentional subcutaneous injection of brimonidine eye drops in an elderly patient resulting in bradycardia, hypotension, and central nervous system depression that responded to high dose naloxone.^3^ This has not been reported previously and has similar findings to other central alpha-2 adrenergic agonist poisonings.

A case series reported on five paediatric patients suffering from inadvertent ingestion or intranasal administration of brimonidine eye drops, which resulted in bradycardia, apnoea, hypotension, and seizure.^4^ One of these patients required cardiopulmonary resuscitation and one of the apnoeic infants was resistant to naloxone treatment. The authors concluded that brimonidine could potentially be lethal in young infants. A case report outlined a similar presentation in a child presenting with bradycardia after accidental ingestion of brimonidine after using Mirvaso as toothpaste.^5^

The European Medicines Agency issued guidance in terms of the marketing authorization for Mirvaso in 2017.^6^ They referenced six cases of bradycardia related to Mirvaso, albeit with confounding factors in some cases. They concluded that it is biologically plausible for there to be increased systemic absorption of Mirvaso through inflamed skin, which could result in bradycardia. They also identified hypotension, which, along with bradycardia, could be explained by increased alpha-2 stimulation.^6^ Dizziness was observed, possibly because of the aforementioned haemodynamic effects, and added as an uncommon side effect.

It is possible in this case, given the severity of patient’s rosacea, that breaks in the skin barrier may have increased absorption of Mirvaso causing symptoms of light-headedness and dizziness and presenting as bradycardia, hypotension, and syncope. This case highlights the importance of taking a full drug history and considering the side effects of medications given as topical preparations. When using Mirvaso, it is important to avoid application to irritated or damaged skin to minimize the possibility of systemic absorption. It is also important to consider if there is a temporal association between clinical features and medication administration as there appeared to be in this case. The 2021 ESC guidelines on cardiac pacing and cardiac resynchronization therapy issue a class III recommendation that pacing is not recommended in patients with bradyarrhythmias related to sinus node dysfunction that are asymptomatic or due to transient causes that can be corrected and prevented.^7^

## Patient perspective

The patient reported only having dizziness and light-headedness prior to his first hospital admission. He found that in the 3 weeks prior to his first admission, around 1.5–2 h after applying Mirvaso, he would be increasingly drowsy. This episode affected the patient’s well-being, including his fitness to drive.

## Data Availability

The data underlying this article are available in the article and in its online supplementary material.

## Lead author biography

Dr Daniel Jesudason is a junior doctor currently working as a clinical teaching fellow in general medicine in Nottingham, UK. He has completed the Internal Medicine Training programme and has a post-graduate diploma in Preventative Cardiovascular Medicine and aspires to undertake higher speciality training in cardiology. His sub-speciality interests include electrophysiology, heart failure, and imaging.

**Consent:** The authors confirm that written consent for submission and publication of this case report including associated text has been obtained from the patient in line with COPE guidance.

**Funding:** None declared.
